# Improving the Melt Flow Length of Acrylonitrile Butadiene Styrene in Thin-Wall Injection Molding by External Induction Heating with the Assistance of a Rotation Device

**DOI:** 10.3390/polym13142288

**Published:** 2021-07-12

**Authors:** Pham Son Minh, Minh-Tai Le

**Affiliations:** HCMC University of Technology and Education, Hochiminh City 71307, Vietnam; tailm@hcmute.edu.vn

**Keywords:** injection molding, mold heating, dynamic mold temperature control, induction heating, flow length, thin-wall injection molding, cycle time

## Abstract

In injection molding, the temperature control of the dynamic mold is an excellent method for improving the melt flow length, especially of thin-wall products. In this study, the heating efficiency of a novel heating strategy based on induction heating was estimated. With the use of this heating strategy, a molding cycle time similar to the traditional injection molding process could be maintained. In addition, this strategy makes it easier to carry out the heating step due to the separation of the heating position and the mold structure as well as allowing the ease of magnetic control. The results show that, with an initial mold temperature of 30 °C and a gap (G) between the heating surface and the inductor coil of 5 mm, the magnetic heating process can heat the plate to 290 °C within 5 s. However, with a gap of 15 mm, it took up to 8 s to reach 270 °C. According to the measurement results, when the mold heating time during the molding process increased from 0 to 5 s, the flow length increased significantly from 71.5 to 168.1 mm, and the filling percentage of the thin-wall product also increased from 10.2% to 100%. In general, the application of external induction heating (Ex-IH) during the molding cycle resulted in improved melt flow length with minimal increase in the total cycle time, which remained similar to that of the traditional case.

## 1. Introduction

In recent years, the injection molding process has undergone many improvements to satisfy the demand for thinner, smaller products or for molding with a low-flow material. Due to its low cost and the capacity for high-volume production, thin-wall and microinjection molding is used to manufacture a variety of polymer components. Most applications of thin-wall and microinjection molding are in the micro-optics and microfluidic devices market. The development of micro-optical parts of various shapes, such as optical gratings, optical switches, and waveguides [1,2,3,4], as well as a variety of molded microfluidic devices, including pumps, capillary analysis systems, and lab-on-a-chip applications [5,6], is ongoing.

Related to the melt flow in the cavity, the appearance of a frozen layer is the main reason for a reduction in the filling ability. To address this challenge, a number of methods have been tested, with the aim of reducing the filling pressure [7] and the viscosity of the melt material [8,9,10] or increasing the filling speed. When the aim was to increase the filling pressure, a high injection pressure was selected, and the experiment showed that the melt flow length was improved. However, to satisfy the requirements of a high filling pressure and high filling speed, the optimization of the injection molding process still needs to be investigated. In addition, the mold structure should also be given further attention due to the ease with which flash problems can occur. There is some existing research about additives for improving the quality of parts. However, to increase the melt flow length by this method, a higher material cost is incurred; on the other hand, in some cases, the molding material is fitted by the customer. In order to reduce the frozen layer, the use of a high mold temperature has yielded good results in reducing the filling pressure and clearly improving the melt flow length [11,12,13]. According to research on mold temperature control [14,15,16,17,18], this is a crucial aspect of plastic manufacturing in the field of injection molding. The quality of the part is improved with a high mold surface temperature, although with undesirable increases in both the cooling time and the cycle time. Decreasing the temperature of the mold surface minimizes cooling time, but there is no benefit in terms of the surface quality of the product [19,20,21]. In recent research, therefore, it has been noted that a crucial requirement is to raise the temperature of the mold surface while minimizing the cycle time.

To achieve this aim, there are many methods for increasing the cavity temperature. The most popular method is to use a higher-temperature fluid such as hot water or hot oil, which flows inside the cooling channel. This method could control the mold temperature when the target temperature is lower than 100 °C [22,23,24,25]. When the heating target is higher than 100 °C, local mold heating with electric heaters has been suggested [25]. In addition, some heating methods were suggested such as heater heating [17,26,27] and steam heating [28,29]. Both methods involve hot fluid flow inside the cooling channel and heater heating such that the core or cavity plate is heated. This is a disadvantage of these heating methods, which leads to a low heating rate as well as energy wastage. Therefore, to address this issue, instead of heating the entire volume of the mold plate, recent research has suggested new heating methods in which only the cavity surface is heated. To achieve this, many methods for mold heating have been suggested, such as hot gas heating [30,31,32,33], induction heating [34,35], and infrared heating [36,37,38]. These methods could support high mold temperatures for improving the melt flow length by reducing the amount of frozen layer formed during melt flow. However, despite achieving the target of reducing both the heating time and thermal energy wastage, the heating time was not adequately minimized. In general, when raising the cavity surface temperature to that of the glass temperature of the plastic material, the required heating time is around 10 s or longer [31,32,33,34,35,36,37,38]. This means that the molding cycle time is longer than the traditional cycle of around 10 s, i.e., it significantly exceeds 10 s.

To address this disadvantage, this paper suggests a new strategy for raising the cavity temperature using an induction heating method. Induction heating has many benefits, including a quick heating time, low energy consumption, and reduced emissions. In this strategy, the heating step is carried out with a cavity insert during the molding process operation, so that the cycle time is not significantly impacted by the heating time. In this study, the gap between the induction coil and the cavity insert was varied from 5 to 15 mm and the heating time was varied from 1 to 8 s. The heating was observed by simulation with COMSOL software; then, the simulation results were collected and compared with experimental results to verify their accuracy. After this, the heating process was applied for real injection molding, while observing the melt flow length and the filling ability of the thin-wall product. In this research, for improving the flow ability of ABS (acrylonitrile butadiene styrene) melt when it is filled into the thin-wall cavity, the molding was achieved with ABS material from Kumho Petrochemical, Seoul, Korea. For the molding experiment, the different targets of mold temperature were achieved, and the filling ability of the material collected and discussed.

## 2. Simulation and Experimental Method

### 2.1. The External Induction Heating with the Assistance of a Rotation Device

External induction heating with a rotational structure for mold temperature control (Ex-IH) is a new technique that can directly and rapidly heat the surface of a cavity insert during the process of injection molding. The external induction heating (Ex-IH) device used in this research consists of an induction heating unit and a rotational structure. The role of the induction heating system is to provide a heat source, which will transfer heat to the cavity insert’s surface and raise the temperature. In this research, the Eagle Fly Induction heating source from X-Forming Company in Hochiminh City, Vietnam, was applied; this heating source can support a maximum current of up to 750 A, and the highest frequency is 75 kHz. In addition, a mold temperature controller was used for the coolant device to provide the cooling fluid at a given temperature to cool the mold after the filling process and to heat the mold to the initial temperature at the start of the experiment.

**Figure 1:**
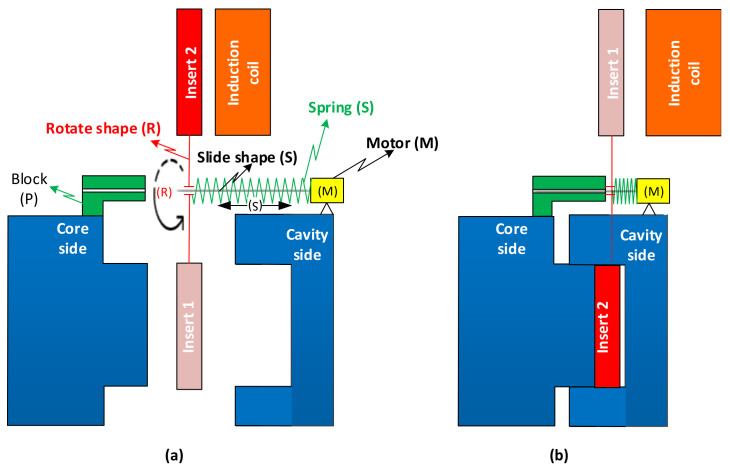
The principle of rotation structure with the position of mold open (**a**) and mold closed (**b**).

In this research, two inserts are used for controlling the cavity temperature. These inserts are changed every molding cycle by the rotation structure as Figure 1a. This structure includes two insert plates, a rotational shape (R), a slide shape (S), a spring, and a motor (M). Two inserts are assembled on the two sides of rotational shape. For changing the position of these inserts, this shape rotates around the center line of slide shape by receiving the rotation moment from motor (M). At the initial position, when the mold opens, the position of two inserts and rotational structure are the same as in Figure 1a. When the mold closes, the core plate moves to the cavity side, and the block presses the rotational shape to the cavity side; therefore, the rotational shape and the two inserts move to the cavity side. This moving is finished when the mold totally closes, as in Figure 1b. When the molding cycle finishes, the two-mold half opens. At this step, the core plate moves to the left side, and the mold plates returns to the position shown in Figure 1a. In this step, the spring (S) presses the rotational shape, and it slides to the farther side of the cavity plate, and the two inserts are rotated to change their positions for the next cycle.

**Figure 2:**
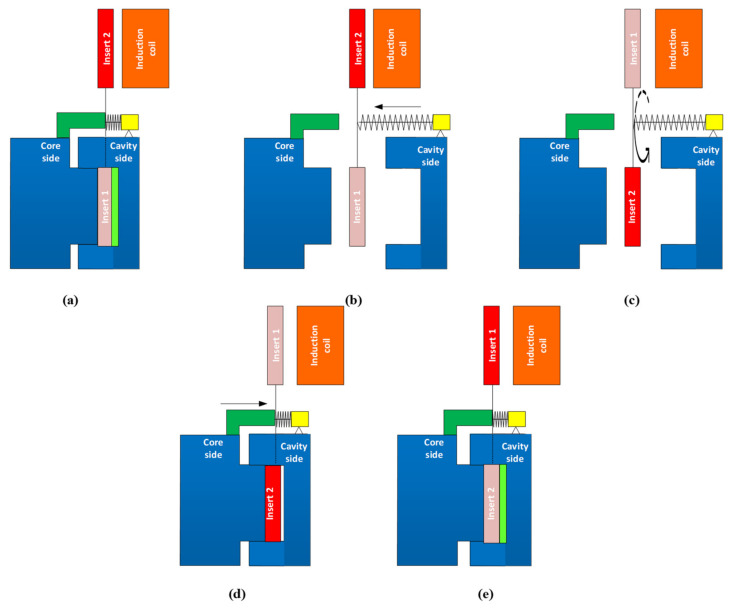
Mold steps in the heating stage of Ex-IH process: (**a**) at the end of molding process; (**b**) mold opening for insert changing; (**c**) the rotation device changes the location of hot and cool insert; (**d**) mold closing with the high-temperature insert inside the cavity; and (**e**) the molding starts as the traditional molding process.

In order to apply the Ex-IH to the molding cycle, the following steps were used: first, the induction heater was used as a heating source for heating insert 2 of the injection molding system. The induction heater produces high-frequency currents in the coils. This current generates a magnetic field of the same frequency as the high-frequency source, which varies around the coil. When a high-frequency current is transmitted through the coil, a high-frequency magnetic field with variable frequency is produced, and an eddy current appears on the insert’s surface. This current heats the insert’s surface. This heating step is performed during the molding cycle as in Figure 2a—Step 1. Second, when the heating step and the molding cycle are complete, the two half mold plates open as in Figure 2b—Step 2. In this step, the plastic product is ejected in preparation for the new molding cycle. Simultaneously, the rotation system is operated, and the locations of inserts 1 and 2 are changed together. The new positions of these inserts are shown in Figure 2c—Step 3. In this step, the high-temperature insert is located on the inside of the molding area in preparation for the new molding cycle, and the lower-temperature insert is located on the outside of the molding area in preparation for the new heating step. After this, the two half mold plates move to the closing position as in Figure 2d—Step 4. After this, the melt is pressed into the molding cavity to form the new plastic product. In this step, due to the melt flow’s contact with the high-temperature area of insert 2, the frozen layer is reduced; in this way, the filling ability can be improved.

### 2.2. Simulation Method

**Figure 3:**
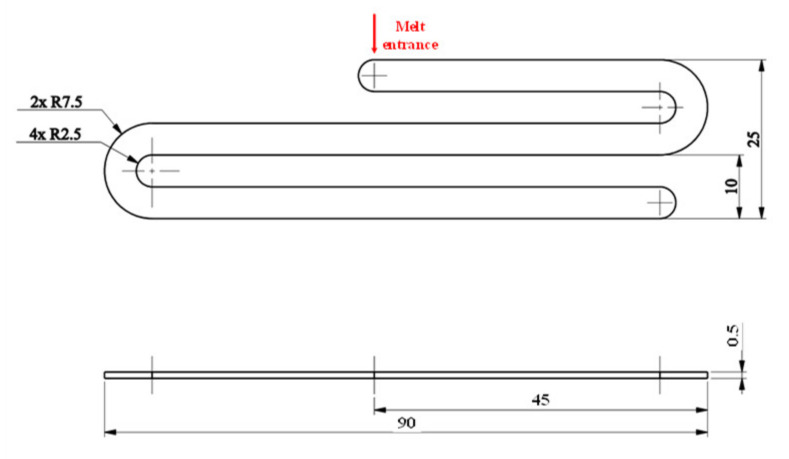
The dimensions of the flow length model.

**Figure 4:**
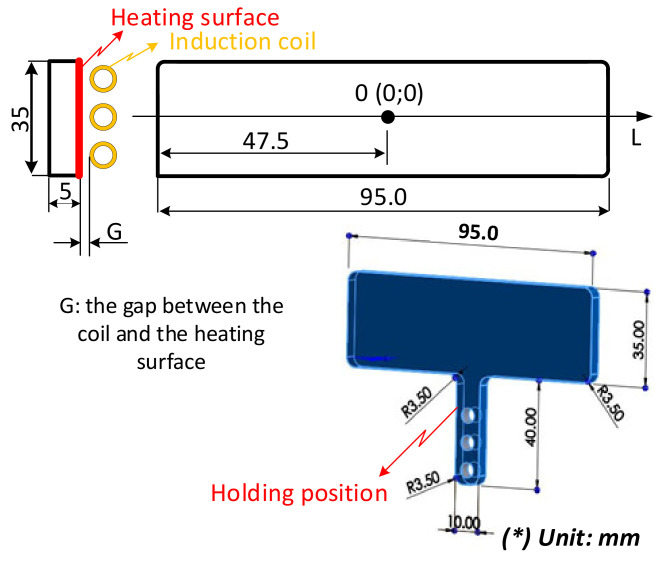
Insert plate dimensions and the gap between the induction coil and heating surface.

In this research, to observe the melt flow length of acrylonitrile butadiene styrene (ABS) in thin-wall injection molding with the assistance of external induction heating, a melt flow length model was built as in Figure 3. In this model, the melt flows into the thin cavity with a thickness of 0.5 mm and a width of 10 mm. The entire size of the cavity area is 25 mm × 90 mm. Therefore, to create a high cavity temperature and reduce the amount of formed frozen layer, two inserts with the same size were designed and manufactured as in Figure 4. These inserts’ design has a width of 35 mm and a length of 95 mm. The insert thickness was selected to be 5 mm. According to other studies [34,35], a thinner insert will support a higher heating speed; however, in such a heating strategy, the insert is rotated after the molding cycle is finished; therefore, a thinner insert reduces the rigidity of the rotation system. In addition, because the induction heating method only impacts the insert surface during the heating period, the thickness of the insert does not significantly impact the heating speed in this case. Therefore, a thick insert was selected for improving the stability of the rotation system. Figure 1b also shows the position of the insert and the induction coil during the heating period. In other studies [11,35], the gap between the induction coil and the heating surface was found to be an important parameter affecting the heating speed and the temperature distribution of the heating surface. However, in other research, the induction coil heats the cavity surface directly when the two half mold plates are open, so the space in which the coil can move, and the heat is limited. In this research, the heating position is located outside of the molding area, so the distance between the coil and the heating surface can be easily established. In addition, in traditional induction heating of the mold surface, the temperature as well as the heating area are harder to control due to the impact of magnetic forces on the ferric material. On the contrary, in the heating strategy presented in this paper, the heating position is separated from the mold plates; therefore, the magnetic control of the heating is much easier. In this research, to observe the influence of the gap between the induction coil and the heating surface as well as the temperature distribution, this gap was varied from 5 to 15 mm in the simulation and experiment.

**Figure 5:**
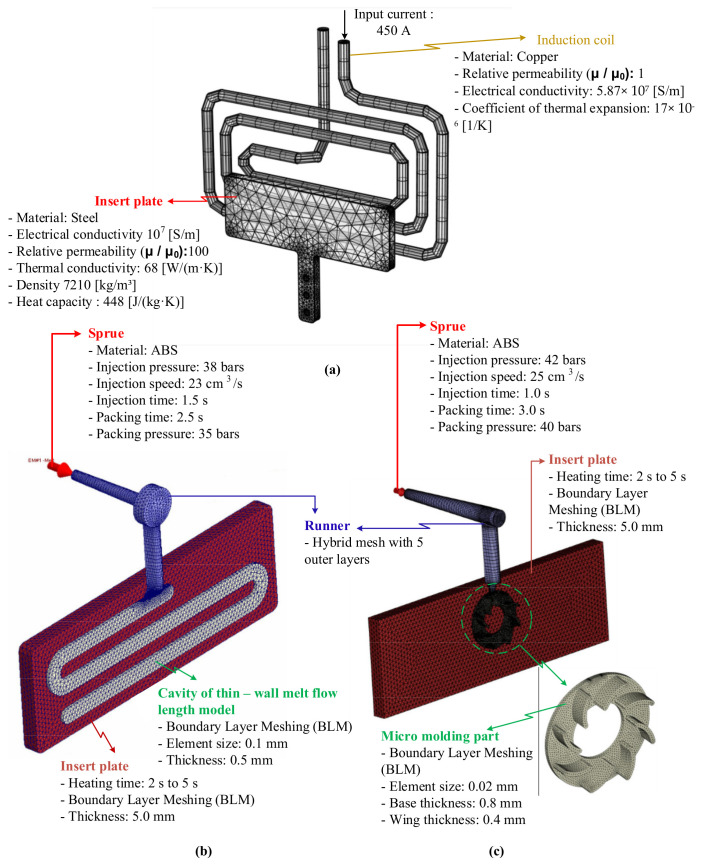
Meshing model for the simulation process in induction heating step (**a**) and molding with the model of melt flow length (**b**) and micro product (**c**).

In the field of mold temperature control, one of the advantages of induction heating is the ability to predict the heating result [11,34,35]. However, the heating position in this research is different from that of the traditional induction heating; therefore, the heating process was achieved using the meshing model as in Figure 5a and Table 1. In this model, the coil material is copper, and the insert plate is steel material. The main parameters for heating simulation of copper and steel are shown in Figure 5a. The coil has a diameter of 8.0 mm. The dimension of insert plate is the same with Figure 4. During simulation, the heat transfer mode around all external surfaces of the insert plate was set at free convection to the air, with an ambient temperature of 30 °C and a heat transfer coefficient of 10 W/m^2^ K. To improve the simulation result, the insert plate was meshed by a triangular mesh, and the corner refinement method was applied for the corner positions. In addition, to reduce the simulation time, the coil was meshed using a 3D swept mesh, which could provide faster calculation. The meshing model and the boundary conditions were imported into COMSOL software (Pitotech Co. Ltd., Chang Hua City, Taiwan) for running the simulation.

In this study, for observing the improving of the filling ability, the model of melt flow length testing was designed, and the meshing model was built as in Figure 5b as a simulating step. In addition, the application of Ex-IH on the micro molding part was also simulated with the meshing model as in Figure 5c. These simulation models include the runner system, molding part, and the insert plate. The runner system has the melt entrance with the parameter as in Figure 5b,c. The hybrid mesh with 5 outer layers was applied for the runner meshes. The melt flow length cavity and the micro molding part were meshed by the boundary layer meshing (BLM) with the element size of 0.1 mm and 0.02 mm, respectively. In this research, to observe the influence of Ex-IH on the filling ability of hot melt, the model of insert plate was added into the simulation model. In the simulation process, the insert plate is set at the temperature with the heating time varied from 2 to 5 s. For running the simulation process, the Moldex3D software (CoreTech System Co., Ltd., Chupei City, Hsinchu County 302, Taiwan) was used with the function of filling.

### 2.3. Experiment Method

To observe the influence of external induction heating on the injection molding process, the real molding process of a plastic product as an insert block was used for this experiment. With the common injection molding process, this type of product involves a kind of thin-wall injection molding. With this product type, the issues with short shot are readily encountered if the injection pressure is low. However, when the injection pressure is too high, problems with flash can easily occur. Therefore, due to its ability to control mold temperature, external induction heating was applied for this molding process and expected for improvements in the product flow length when the injection molding process was operated with a moderate injection pressure.

**Figure 6:**
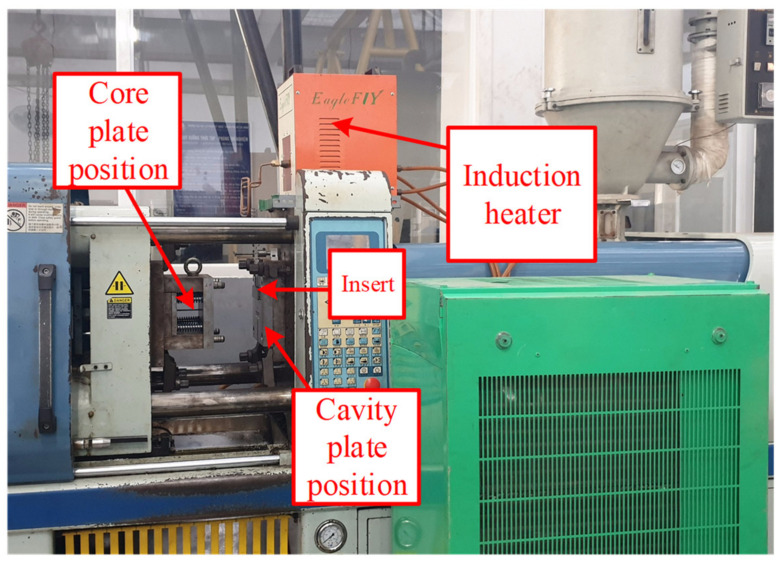
The experiment model for Ex-IH.

**Figure 7:**
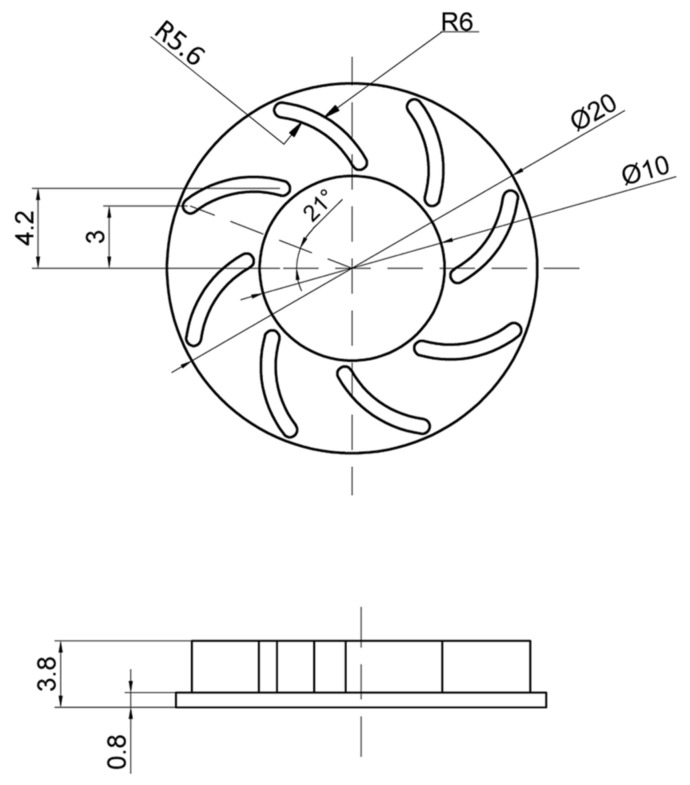
The dimensions of microproduct part.

**Figure 8:**
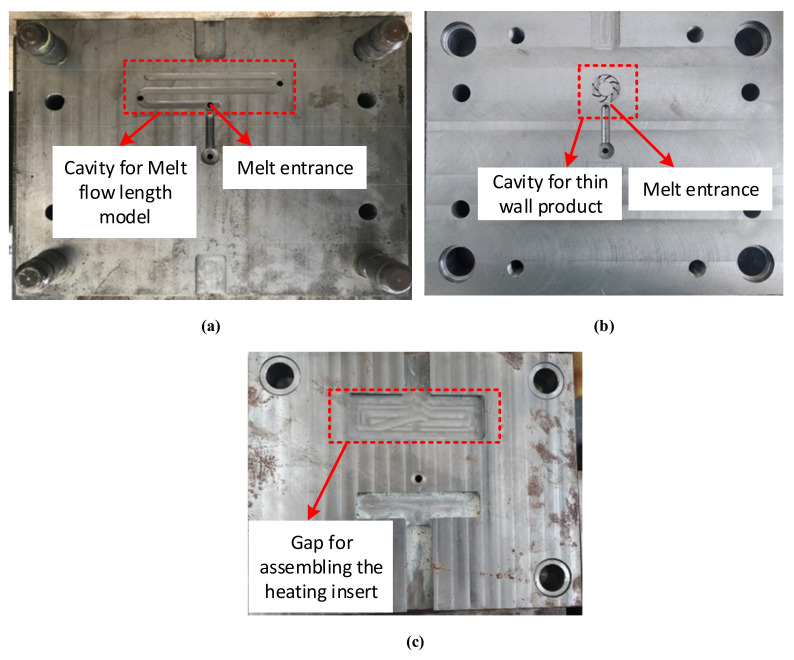
The cavity plate for (**a**) the melt flow length model, (**b**) thin-wall product, and (**c**) the core plate with the gap for assembling the heating insert.

In injection molding field, ABS (acrylonitrile butadiene styrene) is a popular material that provides favorable mechanical properties such as impact resistance, toughness, and rigidity when compared with other common polymers. In the molding process, the molding temperature impacts the final properties of ABS product. For example, molding at a high temperature improves the gloss and heat resistance of the product, whereas the highest impact resistance and strength are obtained by molding at low temperature. ABS is one of many types of thermoplastics with biomedical applications, with injection-molded components being easy to manufacture for single use. In addition, ABS is also a popular material for microparts in the electrical industry. In general, ABS has a wide application in industry; however, one of the highest challengers for producing is the shaping ability of ABS, especially with the thin-wall product. Therefore, in this study, the plastic material of ABS (acrylonitrile butadiene styrene) is used for the molding process, and the molding parameters are maintained for all testing cases. In the experiment, the molding machine of SW-120B (Shine Well Machinery Co., Ltd., Tai-Chung City, Taiwan) is used. The Ex-IH system, the mold, and mold temperature control were connected as in Figure 6. For estimating the influence of Ex-IH on the filling ability of the thin-wall injection molding product, the plastic product shown in Figure 7 was used for testing. This product has a base thickness of 0.8 mm and a wing thickness of 0.4 mm. The insert for the thin-wall product was selected as the subject of the melt flow length model, which is introduced in Figure 4. The mold plates used for the experiment are shown in Figure 8. The molding process was achieved with the parameters presented in Table 2. After the molding was finished, the product was collected and measured by ATOS Compact Scan 2M (GOM GmbH company, Schmitzstraβe, Braunschweig, Germany). The results of the flow length and filling percentage are compared and discussed.

In this study, for observing the temperature distribution of the insert plate under different heating parameters after the heating process was finished, a Fluke TiS20 infrared camera (Fluke Corporation, Everett, Washington, DC, USA) was used for capturing the temperature distribution at the heating surface. The temperature distribution was observed at the time that the insert was moved to the position shown in Figure 2c, immediately prior to filling. Therefore, there was a delay between the end of heating and the point of observation. This delay is around 3 s. Thus, in this study, the collected temperature distribution does not reflect the result at the end of the heating time. This temperature distribution is close to the temperature distribution at which the hot melt is contacted.

## 3. Results and Discussion

### 3.1. Effect of the Gap between the Heating Surface and the Induction Coil

In this research, due to fact that the heating position is located on the outside of the mold structure, the heating step was not significantly impacted by other parts. In addition, this heating strategy could provide a free volume for setting up other devices for controlling the magnetic flux [39], which will help to improve the heating efficiency. For the heating step in this investigation, the heating process was carried out by the coil and the insert, with their positions shown in Figure 5a and Figure 6. In traditional induction heating for injection molding, the gap between the coil and the heating surface is an important parameter. A small gap could support a higher heating speed, reducing the heating time. However, with a small gap, the coil and the heating surface could come into contact, and the cavity surface may thus be damaged. In addition, because the plastic melt temperature is lower than 300 °C, the temperature limit of the insert should be researched for the case when the temperature range is lower than 300 °C.

For observing the influence of the gap between the heating surface and the induction coil, the heating process was simulated, and the gap between the heating surface and induction coil was varied from 5 to 15 mm. After this, the data for temperature distribution of the insert surface and the temperature at point O (as in Figure 4) were collected and compared.

**Figure 9:**
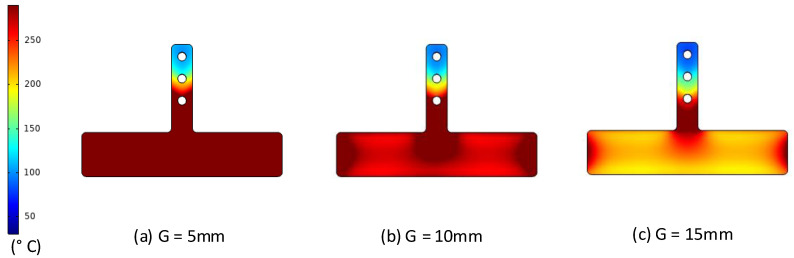
Simulated temperature distribution of insert (in °C) with a heating time of 5 s and a gap (G) of (**a**) 5; (**b**) 10; and (**c**) 15 mm.

**Figure 10:**
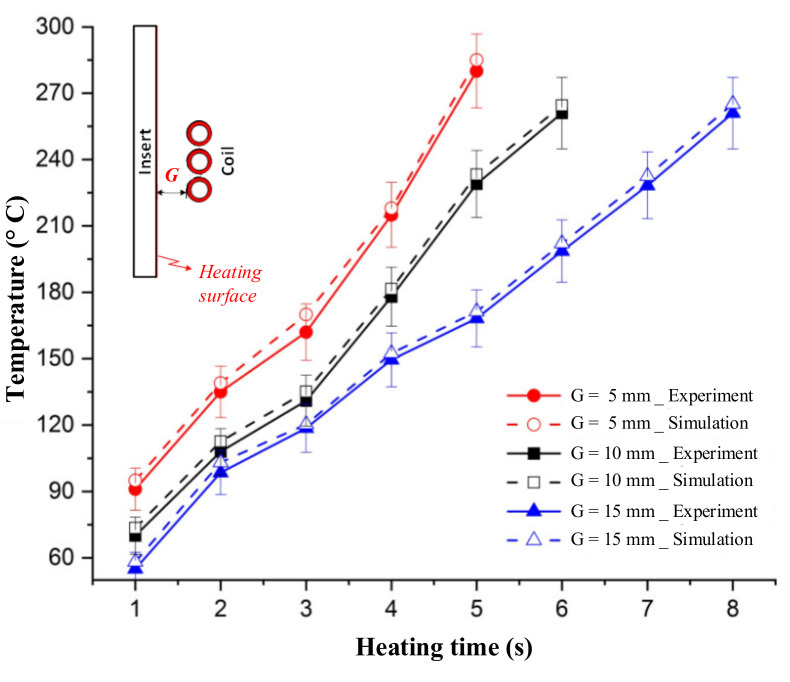
Comparison of temperature history at point O between experiment and simulation.

The variation in the mold temperature with distance is described in Figure 9 and Figure 10. In the simulation, for an initial mold temperature of 30 °C and a gap (G) of 5 mm, it can be seen that the magnetic heating process can heat the plate to 290 °C in 5 s. However, at the distance of 15 mm, it took up to 8 s to reach 270 °C. In general, the shorter the distance, the stronger its influence on the heated plate. Thus, with a shorter distance, a higher heating rate is achieved at the measuring point. This result could be observed clearly during the simulation and experiment, with the temperature distribution shown in Figure 9. For real application in the molding cycle, the result of heating time shows that this heating strategy has almost no impact on the cycle time, which is often varied from around 10 to 20 s. Therefore, depending on the cycle time, the gap (G) could be set to the greatest value needed to ensure that the heating rate is not too high and maintain the safety of the coil and the insert surface. This result also shows that this heating method is appropriate for the insert, which can easily reach temperatures over 200 °C. Figure 10 also shows that the simulation and experimental results are nearly equal, indicating that the actual results are reliable.

### 3.2. Effect of the Heating Time on the Temperature Distribution

**Figure 11:**
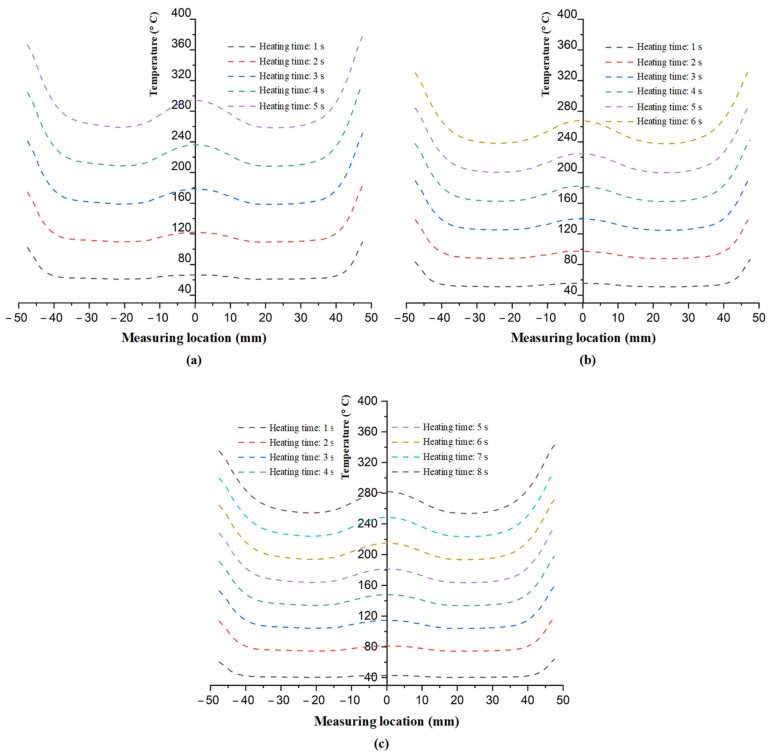
Simulation result of temperature distribution of line L with the gap (G) of (**a**) 5; (**b**) 10; and (**c**) 15 mm at the end of heating time.

As mentioned in Figure 2, in this study, because the heating process takes place during the molding cycle, the heating time simply needs to be shorter than the molding cycle; therefore, the total time needed for one cycle should not be longer than in cases of traditional molding. In addition, a slower heating rate could allow for a longer working lifetime of the insert due the reduction in residual stress when the insert temperature increases. Thus, in this study, the heating time was observed with values varying from 1 to 8 s, with the heating gap varied from 5 to 15 mm. The temperature data for line L (as in Figure 4) were collected by simulation and experiment. The variation in the mold temperature of line L versus heating time is described in Figure 11. This result shows that the temperature of line L increased clearly with the longer heating time. In addition, the different temperatures of line L clearly show the influence of edge effect. Due to the edge effect, the temperature on two sides of the insert rapidly increased. In addition, this effect also allowed the temperature of the holding area to rapidly increase; this result could be observed clearly in Figure 9c. Therefore, this is the reason for the higher temperature in the central area of line L. Figure 11 also shows that overheating could occur at the side of the insert. This is also a disadvantage of the induction heating method in the injection molding field. The result shown in Figure 11 also demonstrates that a lower heating rate could mitigate the edge effect. Figure 11a shows that the heating time of 5 s supports the temperature at the central point of over 280 °C when the temperature at the two edges is over 360 °C. However, with a slower heating rate, Figure 11c shows that the central temperature could reach 280 °C, but the side temperature is lower than 360 °C, which is around 340 °C. Thus, this result demonstrates that the edge effect of induction heating could be reduced by using a lower heating rate or with a longer heating time. This is another advantage of Ex-IH, which could support a longer heating period than that of the traditional induction heating process.

**Figure 12:**
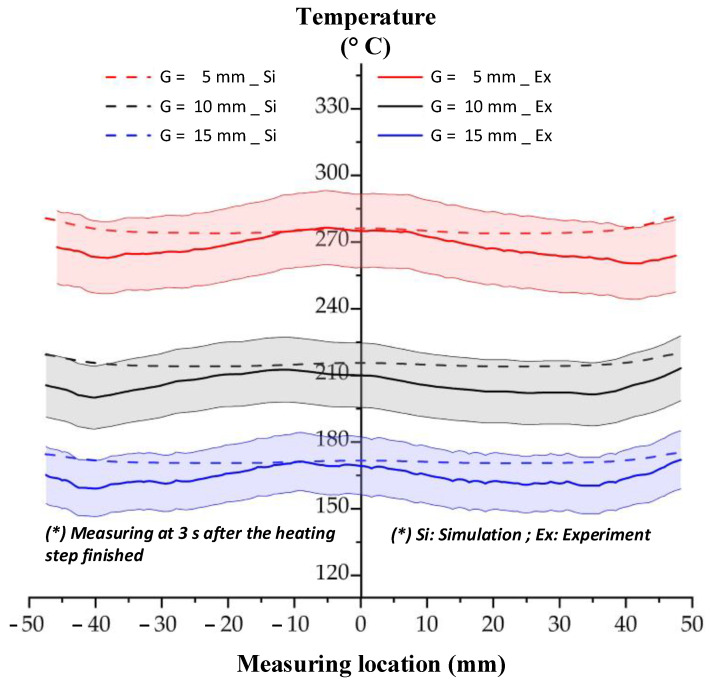
Comparing the temperature distribution of line L between simulation and experiment with the heating time of 5 s.

For verifying the simulation result, the experiment was performed with the same boundary conditions as the simulation. The temperature information of line L was collected by an infrared camera. In experiment, when the heating process finished, the insert plate needed about 3 s for rotating to the molding position; therefore, the temperature at this time was collected. In addition, in simulation, the temperature distribution was also collected at 3 s after the end of heating step. The comparison between the simulation and experiment is shown in Figure 12. Compared with Figure 11, this result shows that the temperature profile of line L undergoes a change after 3 s, when heating is complete. The temperature was more uniform, and the high temperature at the two sides was clearly reduced due to the heat transfer from the higher temperature to the lower temperature. With the heating time of 5 s, the experimental result shows that the temperature of line L varied around 280.0, 210.0, and 168.0 °C with a gap of 5, 10, and 15 mm, respectively. This result also demonstrates that the Ex-IH could support the heating process for the cavity area of 35 mm × 95 mm and has strong potential for application in the field of mold temperature control.

### 3.3. Improving the Melt Flow Length of Front Cover Part by External Induction Heating for the Gate Temperature Control

For observing the influence of Ex-IH on improving the melt flow length, the testing model and real thin-wall product were designed as in Figure 3 and Figure 7. For the experiment, the injection mold was designed and manufactured as in Figure 8. The substance to be melted was ABS. For both models, the heating time was varied from 2 to 5 s with a gap (G) of 5 mm. Nonetheless, for complete filling of the cavity, the mold temperature must be set to the highest possible value for the device with the thin-wall product, as in this case. Due to the reduction in the freeze layer of the melt flow, the hot melt flows easier. However, when the mold temperature is at a high value, energy wastage occurs along with other issues such as warpage and flashing. In this paper, control of the mold local temperature was specifically discussed to minimize these problems. Instead of keeping the entire mold plate at a high temperature, the local mold temperature was controlled at the beginning of the molding process by Ex-IH. The high temperature at the core side reduces the melt flow pressure drop as it flows over the area. Figure 8 shows the plate of the cavity, which includes the region of the cavity and of the gates.

**Figure 13:**
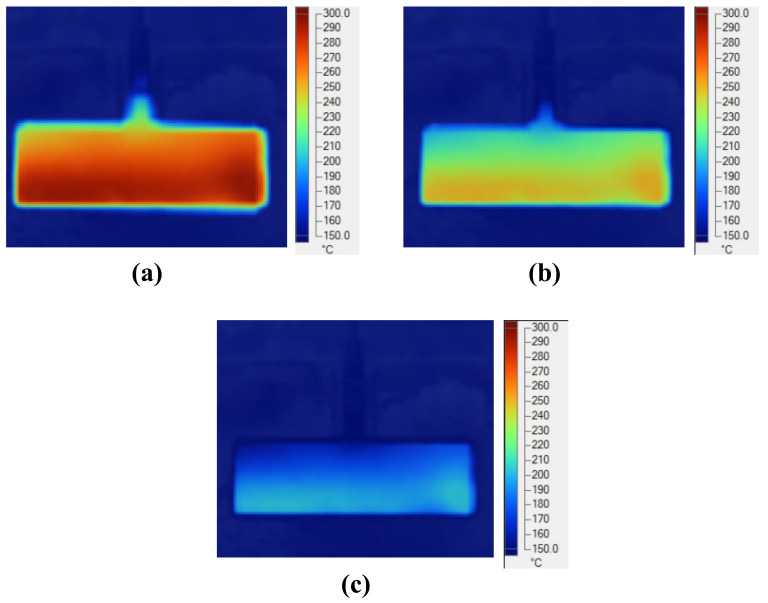
Measured temperature distribution of cavity plate after 5 s heating with the gap (G) of (**a**) 5; (**b**) 10; and (**c**) 15 mm.

For observing the effect of high-frequency magnetic forces on the heating method, an infrared camera was used to capture the temperature distribution at the end of the heating step to verify the heating efficiency as well as the capacity of the local heating. The result of the product heating test at different distances at 5 s is shown in Figure 13. This result shows that the temperature distribution is focused at the insert area; this distribution is almost the same as the simulation result, which is shown in Figure 9.

**Figure 14:**
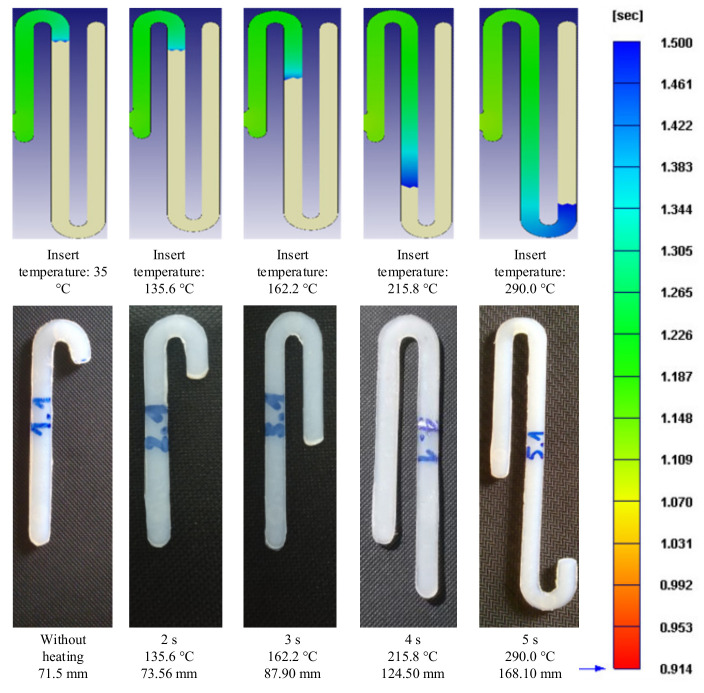
The simulation and experiment of melt flow length model after molding with Ex-IH under different heating times.

**Figure 15:**
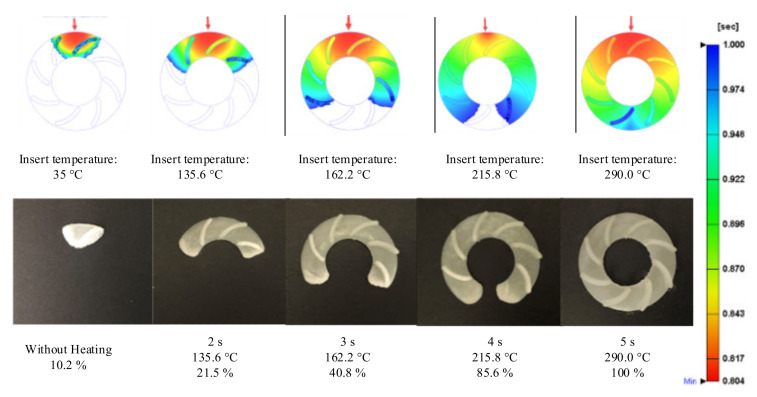
The simulation and experiment of thin-wall parts after molding with Ex-IH under different heating times.

**Figure 16:**
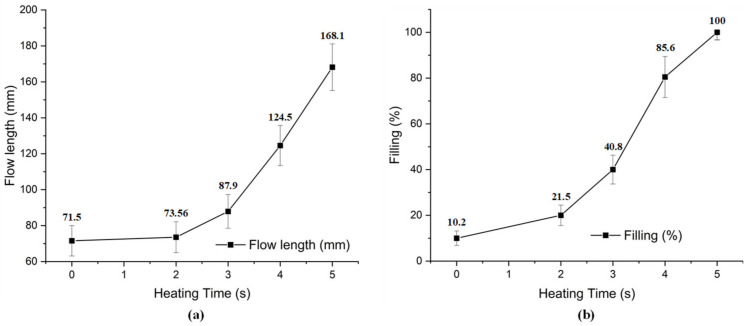
The improvement of melt flow length (**a**) and filling percentage of micro molding product (**b**) when the Ex-IH was applied.

For observing the improving of melt flow length with the assisted if Ex-IH, the simulation was conducted with the meshing model as in Figure 5b,c. In the experiment, the molding samples were collected for observing the improvement of melt flow length under different heating times. The molding samples and the simulation results are shown in Figure 14 and Figure 15. The melt flow length and the filling percentage of thin-wall product were measured and compared as in Figure 16. According to simulation and experiment results, when the mold heating time increases from 0 to 5 s, the flow length increases significantly from 71.5 to 168.1 mm (Figure 14). This means that the Ex-IH improved the melt flow length by around 2.3 times. Figure 14 and Figure 16a show that the melt flow length was increased clearly in the case of 3, 4, and 5 s heating time. This means that with the ABS material, the melt flow length improves when the insert temperature is higher than 162.5 °C. For applying the Ex-IH for real product, the microproduct with the dimension as Figure 7 was applied. The molding was achieved with the mold plate as in Figure 8. The molding was operated in the case of without heating step; then, the Ex-IH was applied with the heating time varied from 2 to 5 s. The molding products were collected and compared with simulation results as in Figure 15. This result also shows that the Ex-IH has a strong influence on the filling ability of microinjection molding part, which was improved from 21.5% to 100% under the heating time increases from 2 to 5 s. This result is a signifiable improvement in injection molding when we compared with the traditional process with the case of without heating step. According to the experiment result, if we increase any temperature (insert sheet temperature or plastic temperature), the flow length and the filling percentage of the product have a strong influence. In general, both models of melt flow length and micro molding product show that the Ex-IH could improve the melt flow length, and the results of simulation and experiment have a good agreement.

## 4. Conclusions

In this research, external induction heating with a rotational structure for mold temperature control (Ex-IH) was applied during the injection molding cycle for improving the filling ability. The simulation and experiment were performed focusing on the melt flow length mold and thin-rib molding. For the molding of the melt flow volume, the heating time was varied from 1 to 5 s, and the heating process was then conducted with a gap (G) of 5, 10, and 15 mm. With the thin-wall product, the mold temperature control with the Ex-IH was achieved with a gap of 5 mm, and the heating time increased from 2 to 5 s. According to the simulation and experimental results, the following conclusions were obtained:

- For an initial mold temperature of 30 °C and a gap (G) of 5 mm, it can be seen that the magnetic heating process can heat the plate to 290 °C in 5 s. However, at a distance of 15 mm, it took up to 8 s to reach 270 °C. The heating time results show that this heating strategy has almost no impact on the cycle time, which often varied from around 10 to 20 s. Therefore, depending on the cycle time, the gap (G) could be set to the greatest possible value to ensure that the heating rate is not too high and to prolong the lifetime of the coil and the insert surface.
- The temperature of line L clearly increased with a longer heating time. Due to the edge effect, the temperature on two sides of the insert quickly increased. In addition, this effect also allowed the temperature at the holding area to increase quickly. Varying the heating time from 1 to 8 s, the result shows that a lower heating rate could reduce overheating at the edge of the insert plate.
- The temperature profile of line L undergoes a change after the heating for 3 s is completed. The temperature was more uniform, and the high temperature at the two sides was clearly reduced due to the heat transfer from the higher temperature to the lower temperature. With the heating time of 5 s, the experimental results show that the temperature of line L varies around 168.0, 210, and 280 °C with a gap of 5, 10, and 15 mm, respectively. This result also demonstrates that Ex-IH could support the heating process for a cavity area of 35 mm × 95 mm and has great potential for application in the field of mold temperature control.
- According to the measurement results, when the mold heating time was increased from 0 to 5 s during the molding process, the flow length significantly increased from 71.5 to 168.1 mm, and the filling percentage of the thin-wall product also increased from 10.2% to 100%. In general, when the Ex-IH was applied during the molding cycle, the total cycle time was similar to that in the traditional case.

## Figures and Tables

**Table 1:** Material properties.

Material	Property	Value	Unit
Copper	Relative permeability (μ/μ_0_)	1	1
	Electrical conductivity	5.87 × 10^7^	S/m
	Coefficient of thermal expansion	17 × 10^−6^	1/K
	Heat capacity at constant pressure	387	J/(kg·K)
	Density	8940	kg/m^3^
	Thermal conductivity	398	W/(m·K)
	Young’s modulus	128 × 10^9^	Pa
	Poisson’s ratio	0.34	1
	Reference resistivity	1.72 × 10^−8^	Ω·m
	Resistivity temperature coefficient	3.9 × 10^−3^	1/K
	Reference temperature	273.15	K
Steel	Electrical conductivity	1 × 10^7^	S/m
	Relative permeability (μ/μ_0_)	100	1
	Thermal conductivity	68	W/(m·K)
	Density	7210	kg/m^3^
	Heat capacity at constant pressure	448	J/(kg·K)

**Table 2:** The molding parameters for the product of melt flow length testing and thin-wall product.

Molding Parameters	Unit	Melt Flow Length Testing	Thin-Wall Product
Injection speed	cm^3^/sec	23	25
Injection pressure	Bar	38	42
Injection time	s	1.5	1.0
Packing time	s	2.5	3.0
Packing pressure	Bar	35	40
Cooling time	s	12	
Initial mold temperature	°C	35	
Melt temperature	°C	260	270
Preheating time by Ex-IH	s	2–5	

## Data Availability

The data used to support the findings of this study are available from the corresponding author upon request.
